# *In cellulo* structure determination of a novel cypovirus polyhedrin

**DOI:** 10.1107/S1399004714004714

**Published:** 2014-04-30

**Authors:** Danny Axford, Xiaoyun Ji, David I. Stuart, Geoff Sutton

**Affiliations:** a Diamond Light Source Ltd, Harwell Oxford, Didcot OX11 0DE, England; b Division of Structural Biology, The Welcome Trust Centre for Human Genetics, University of Oxford, Oxford OX3 7BN, England

**Keywords:** microcrystals, viral protein, data collection, *in cellulo*

## Abstract

This work demonstrates that with the use of a microfocus synchrotron beam the structure of a novel viral polyhedrin could be successfully determined from microcrystals within cells, removing the preparatory step of sample isolation and maintaining a favourable biological environment. The data obtained are of high quality, comparable to that obtained from isolated crystals, and enabled a facile structure determination. A small but significant difference is observed between the unit-cell parameters and the mosaic spread of *in cellulo* and isolated crystals, suggesting that even these robust crystals are adversely affected by removal from the cell.

## Abstract 2

The crystal structure of a previously unsolved type of cypovirus polyhedrin has been determined from data collected directly from frozen live insect cells.

## 1. Introduction   

*In vivo* crystallization, despite producing long-recognized cases of biological self-assembly, has typically been regarded as anomalous behaviour (Doye *et al.*, 2004 ▶) and as such has received relatively little attention in the structural biology community. This is especially remarkable when contrasted with the effort that has gone into *in vitro* protein crystallization for structural studies. Notable biological processes that involve *in vivo* crystallization include protein storage in seeds (Colman *et al.*, 1980 ▶), secretion of the hormone insulin (Dodson & Steiner, 1998 ▶), numerous cases of pathological overexpression (Wang *et al.*, 2012 ▶), toxin production by *Bacillus thuringiensis* (Schnepf *et al.*, 1998 ▶) and encapsulation in certain genera of insect viruses (Anduleit *et al.*, 2005 ▶). Crystallization of heterologous protein has also been seen in Chinese hamster ovary (CHO) cells engineered to overexpress immunoglobulin (Hasegawa *et al.*, 2011 ▶). Another engineered expression system, the baculovirus–Sf9 system, exploits the ability of the virus to induce the expression of the protein required for the production of viral inclusion bodies within insect cells. With the use of this method, *in vivo* crystallization has been seen to occur when the expression cassette encodes a related polyhedrin (Zhou *et al.*, 1998 ▶), a chimera of a polyhedrin and the protein of interest (Ijiri *et al.*, 2009 ▶), and, in isolated cases, polyhedrin-free protein (Fan *et al.*, 1996 ▶; Koopmann *et al.*, 2012 ▶). One limiting factor in the harnessing of *in vivo* crystallization for structural studies is the typically small size that crystals grow to, as constrained by the contents and dimensions of the cell. Recently, *in vivo* protein crystallization has been presented as a suitable method for the production of samples for structural studies with X-ray free-electron lasers (XFELs; Koopmann *et al.*, 2012 ▶). The XFEL technique of serial femtosecond crystallography (SFX) is achieved with a large number of small samples passed sequentially through a pulsed, ultrabrilliant X-ray beam (Chapman *et al.*, 2011 ▶). As a proof of principle of this, structure determination of the non-polyhedrin protein cathepsin from *Trypanosoma brucei* has been accomplished *via* SFX (Redecke *et al.*, 2013 ▶), utilizing baculovirus expression to trigger *in vivo* crystallization, followed by purification of crystals. Microfocus beamlines at third-generation synchrotron sources have also proven to be capable of successful structure solution from *in vivo*-produced polyhedra (Coulibaly *et al.*, 2007 ▶, 2009 ▶; Ji *et al.*, 2010 ▶). In all of these structural studies, *in vivo*-grown crystals have been isolated and purified from the host cells prior to data collection. In this work, we present a novel approach whereby crystals are investigated *in cellulo* by mounting frozen live cells directly in the X-ray beam. We use as an exemplar the successful structure determination of a novel polyhedrin from a cytoplasmic polyhedrosis virus (CPV) which infects the winter moth *Operophtera brumata* from our ongoing studies into polyhedra from insect viruses. Not only does the *in cellulo* approach reduce the time and effort required, it also maintains the samples in a biologically relevant environment, *e.g.* in the presence of other cellular components and under cellular ionic conditions. The workflow enabling the structure determination is both time-efficient and sample-efficient, and has produced high-quality data from significantly fewer crystals of only 4–5 µm in diameter, representing a crystal volume roughly an order of magnitude smaller than that of the crystals used for previous analyses (Coulibaly *et al.*, 2007 ▶, 2009 ▶; Ji *et al.*, 2010 ▶).

## 2. Materials and methods   

### 2.1. Infection/expression   

**Figure 1:**
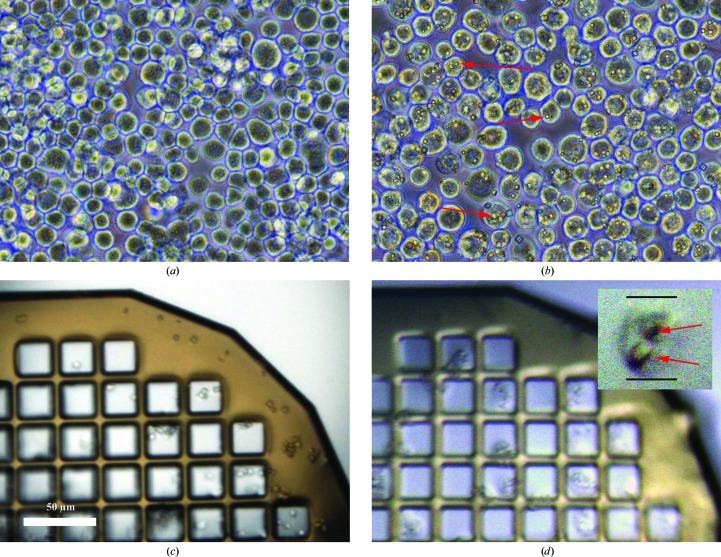
(*a*) Uninfected cells. (*b*) Infected cells. (*c*) Isolated crystals loaded onto a 25 µm mesh, pictured with an on-axis sample view on the beamline during data collection. (*d*) Infected cells loaded onto a 25 µm mesh, pictured with an on-axis sample view on the beamline during data collection; the inset shows a close-up of a single cell with two crystals visible. All panels are shown on the same scale. Red arrows indicate polyhedra crystals.

The polyhedrin gene for *O. brumata* CPV18 (OpbuCPV18) was synthesized by GeneArt (Life Technologies) based on GenBank sequence DQ192250 (Graham *et al.*, 2007 ▶). The polyhedrin gene was amplified and inserted into the transfer vector pBacPAK9 (Clontech). Recombinant baculovirus was produced by co-transfection of linearized baculovirus DNA and the transfer vector following a standard protocol (Zhao *et al.*, 2003 ▶). Expression and purification of polyhedra followed the protocol described in Anduleit *et al.* (2005 ▶). Figs. 1 ▶(*a*) and 1 ▶(*b*) show a comparison between non-infected *Spodoptera frugiperda* (Sf9) cells (Fig. 1 ▶ *a*) and those infected with baculovirus expressing OpbrCPV18 polyhedrin (Fig. 1 ▶ *b*). Polyhedra are clearly visible as white dots in the majority of infected cells.

### 2.2. Data collection   

For *in cellulo* data collection, infected Sf9 cells were harvested 3 d post-infection and concentrated by centrifugation at 43*g* for 90 s. The cells were resuspended in media, mixed in a 1:1 ratio with the cryoprotectant ethylene glycol and allowed to equilibrate for 60 s. Cells in cryoprotectant were applied onto a MicroMesh mount (MiTeGen, Ithaca, USA) and were allowed to settle for 30 s before excess liquid was wicked away. The sample was flash-cooled in a stream of nitrogen gas at 100 K. The isolated crystals were treated with cryoprotectant and cooled in the same way. Examples of isolated crystals and polyhedra within cells cooled on grids imaged on-axis at the beamline are shown in Figs. 1 ▶(*c*) and 1 ▶(*d*). All manipulations of the cells were performed with care so as not to disrupt them. The integrity of the cells was checked at each step and no cell disruption or free polyhedra crystals were observed.

**Figure 2:**
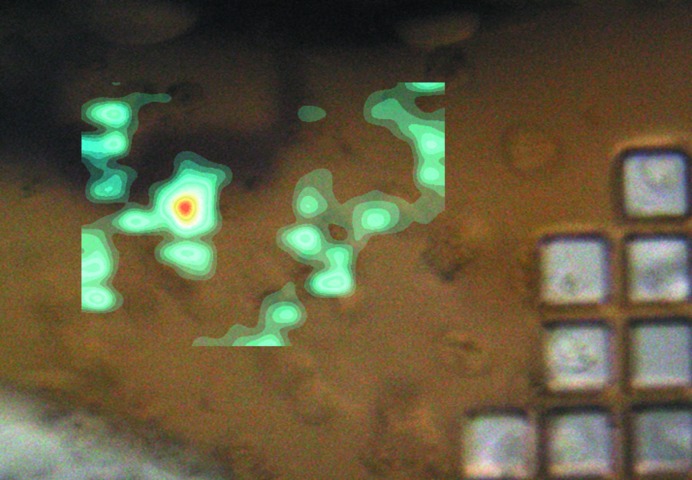
Results of a grid scan from *in cellulo* data collection shown as a contour plot of the *DISTL* output ‘good Bragg candidates’.

The standard optical configuration of the I24 beamline uses two pairs of Kirkpatrick–Baez focusing mirrors: the first accepting monochromatic X-rays and producing a secondary source, and the second further demagnifying the X-ray beam to 10 × 10 µm at the sample with 1.5 × 10^12^ photons s^−1^. However, in this work the best data were obtained with a 6 × 6 µm beam of ∼2 × 10^11^ photons s^−1^ at 12.8 keV, achieved by reducing the size of the secondary source with beam-defining slits, which gave an estimated dose to each crystal of ∼1.4 MGy s^−1^. The detector used to record the *in cellulo* data was a PILATUS3 6M (DECTRIS, Baden, Switzerland). Once mounted onto the sample position, crystals within the cells could not be easily resolved visually, so they were located by fast raster scans of the sample loop (Aishima *et al.*, 2010 ▶). The sample loop was orientated perpendicular to the X-ray beam. An example scan is shown in Fig. 2 ▶, consisting of a 22 × 18 array of 396 points with a spacing of 5 × 5 µm covering ∼10 000 µm^2^. Exposures were still images of 0.2 s with 100% flux and with the edge of detector set to 2.5 Å. Individual frames from the scan were automatically analysed with the software package *DISTL* (Zhang *et al.*, 2006 ▶) and were scored to produce the contour plot. From the results of this scan, 12 data-collection points were selected. At each of these points a sweep of data was collected consisting of 40 images of 0.05° oscillation with an exposure of 0.5 s, with the edge of the detector set to record 1.8 Å Bragg spacing. Seven of these ‘hits’ were included in the final complete data set. In total, three grid scans and 40 wedges, each of 2° of data, were recorded with the spindle rotation range centred on the value used for the grid scan. Each grid scan took around 2.5 min to collect and a total of approximately 75 min elapsed time was required to collect the data used for structure determination.

Data from isolated crystals were recorded at an earlier date with a PILATUS 6M detector installed on the beamline. Although isolated crystals could be seen visually, a similar procedure of crystal detection *via* a grid scan was used to ensure optimum alignment of the sample with the beam. In total, 26 partial data sets of 3° consisting of 30 images with 0.1° oscillations were recorded with an exposure time of 0.25 s per image. A 6 × 6 µm beam size was again used. Data from isolated crystals were processed in a similar way to that described below for the data from cells.

### 2.3. Data processing   

Each partial data set was integrated using the Diamond automated pipeline *FastDP* (G. Winter, unpublished work; Winter & McAuley, 2011 ▶). Data wedges where *FastDP* identified the correct space group (*I*23) were fed to the software package *BLEND* for data merging and scaling (Foadi *et al.*, 2013 ▶). *BLEND* compares the unit-cell parameters across separate partial data sets and clusters data sets on the basis of how isometric they are. *POINTLESS* and *AIMLESS* are used to merge and scale the clustered data and the user is able to compare, on the basis of *R* value and completeness, clusters expanding in size from pairs of data wedges up to the entirety of the integrated data. The resolution cutoff was determined by *AIMLESS* with a CC_1/2_ of >0.5 for the highest resolution shell (Evans, 2011 ▶).

### 2.4. Structure determination and refinement   

Merged and scaled intensities were converted to structure factors with the program *CTRUNCATE*. OpbrCPV18 polyhedrin has 83.5% sequence identity to BmCPV1 (Graham *et al.*, 2007 ▶), making the structure of the latter (PDB entry 2oh6; Coulibaly *et al.*, 2007 ▶) a good search model for molecular replacement using *Phaser* (McCoy *et al.*, 2007 ▶). The model was then rebuilt and refined using *Coot*, *REFMAC* and *PHENIX* (Emsley *et al.*, 2010 ▶; Murshudov *et al.*, 2011 ▶; Adams *et al.*, 2010 ▶). This whole process was performed independently for the data from isolated particles and from crystals in cells.

## 3. Results and discussion   

### 3.1. Crystallographic details of *in cellulo* OpbrCPV18   

**Figure 3:**
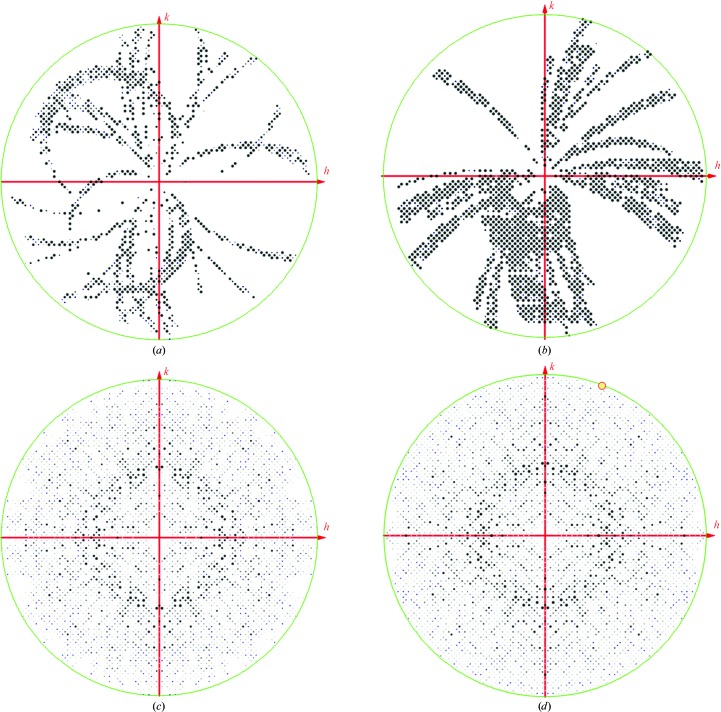
Images of the *hk*0 central sections of intensity-weighted reciprocal lattices. (*a*) and (*b*) show the unreduced indices, whilst (*c*) and (*d*) are after applying crystallographic symmetry. (*a*) and (*c*) are for the *in cellulo* merged data set, while (*b*) and (*d*) are for the isolated crystal merged data set. The images were generated by *ViewHKL* (*CCP*4). The completeness and multiplicity were 91% and 3.7 and 97% and 9.9, respectively.

From a total of 40 *in cellulo* crystals identified from grid scans, 20 were integrated successfully by *FastDP* with the correct space group assigned. Inspection of the images indicated that crystals not achieving automated data reduction were either misaligned (*i.e.* rotating in and out of the beam) or simply diffracted too weakly, with too few observed reflections for correct indexing. There was little indication of multiple lattices. The 20 selected crystals displayed similar unit-cell parameters *a* = *b* = *c* = 103.04 Å, with a standard deviation of just 0.1 Å. *BLEND* was used to compare the unit-cell parameters across separate partial data sets and to cluster data sets assessed to be isometric. *BLEND* then used *POINTLESS* and *AIMLESS* to merge and scale all clusters. The merged data set with the lowest *R*_meas_ with a completeness sufficient for molecular replacement (>90%) was selected. *BLEND* determined that the inclusion of all crystals of a standard sufficient for automatic data integration produced an optimum data set in terms of both completeness and *R* value. Since *BLEND* uses unit-cell parameter similarity as a discriminator for data merging, this reflects the consistency of the measured cell across samples. Fig. 3 ▶ shows that there is no obvious tendency for the crystals to adopt a preferred alignment in the amorphous ice film, although we cannot rule out such an effect for less isometric crystals.

As expected, the structure of OpbrCPV18 polyhedrin is very similar (r.m.s.d. of 0.23 Å for all 248 C^α^ atoms) to that of BmCPV1, although there are several notable differences which will be reported elsewhere (Ji *et al.*, in preparation).

### 3.2. Comparison of *in cellulo* and isolated data   

**Figure 4:**
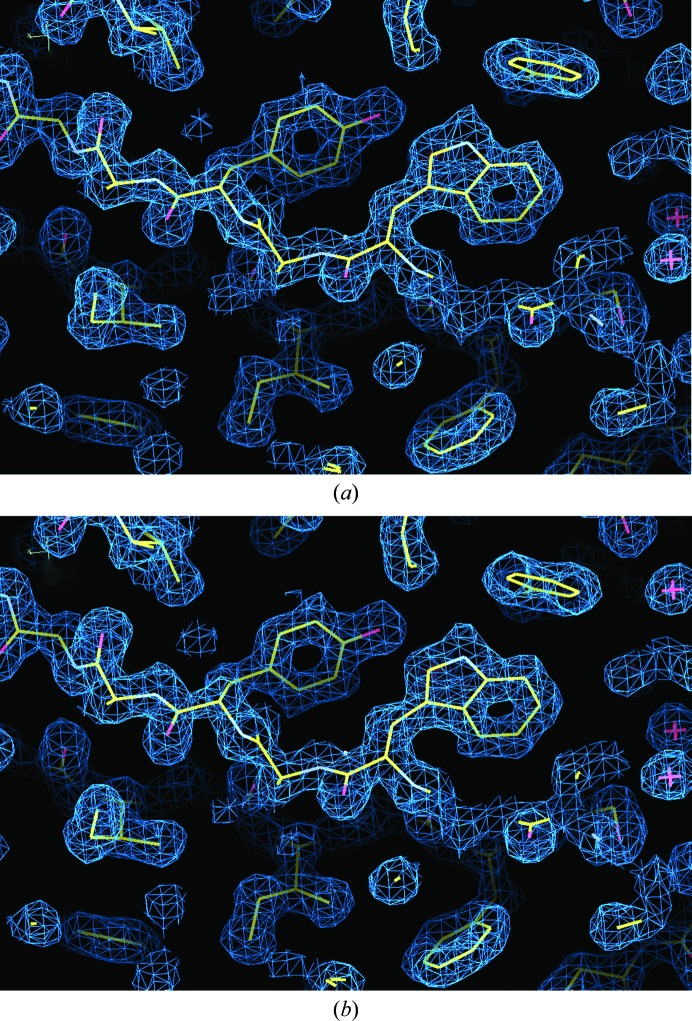
Representative electron density: (*a*) *in cellulo*, (*b*) isolated. Maps shown are 2*F*_o_ − *F*_c_ contoured at 1σ.

Table 1 ▶ compares the two methods of data collection, and there is very little difference in terms of the effective resolution extent and *R*_meas_. The results show no significant reduction in signal-to-noise level in moving from isolated samples to direct measurements within cells, despite the additional cellular material present on the loop. Maps of electron density, shown in Fig. 4 ▶, also support this observation. Overall, the two structures are very similar (r.m.s.d. of 0.07 Å for all 248 C^α^ atoms). The diffraction data do, however, reveal a small difference in both unit-cell parameters and crystal mosaicity between *in cellulo* and isolated crystals. In order to remove the possibility that this difference reflected an artefact from the difference in collection oscillation width (0.05° and 0.1°), the *in cellulo* images were merged in pairs, using the *XDS* tool *merge*2*cbf*, to form equivalent data sets of 0.1° images, which were then treated identically in *XDS* for comparison. The data were only pre-processed in this way for the purposes of this comparison. The isolated crystals display a reduced mean unit-cell parameter of 102.79 Å compared with 103.04 Å for the *in cellulo* crystals and, given the low cell variance, this difference is statistically significant with a *p* value^1^ of 7 × 10^−7^. The mosaicity, as calculated by *XDS*, is slightly higher in the isolated crystals, 0.05° in comparison to 0.03°, and this difference, although not as marked, is statistically significant, with a *p* value of 0.03. The variance of both unit-cell parameters and mosaic spread is higher in the isolated crystals. These observations suggest that removal of the crystals from the regulated environment of the cell induces systematic changes in the crystal lattice, even in these CPV polyhedrin crystals, which are unusually robust (Anduleit *et al.*, 2005 ▶). For the *in cellulo* polyhedrin the compartmentalization of the crystals within the cell means that we do not know the effective concentration of ethylene glycol, and indeed the cellular components may act as the primary cryoprotectant. The slightly worse *R* factor for the *in cellulo* data (Table 2 ▶) reflects the somewhat lower completeness and multiplicity to which the data were measured.

Data were also collected using the full-flux 10 × 10 µm beam. Although we were able to record data of a quality suitable for structure solution, it was clearly inferior to the smaller beam, with a the resolution of the data set reduced from 1.7 to 2.2 Å and *R*_meas_ elevated from 0.19 to 0.32.

## 4. Conclusions and outlook   

We have determined the structure of a novel cypovirus polyhedrin *via in cellulo* crystallography. In a comparison with isolated crystals, the data quality does not suffer significantly, despite the presence of additional material from the cell. Notwithstanding the recognized robustness of CPV crystals, we observe a statistically significant difference in calculated unit-cell parameters and mosaicity between *in cellulo* and isolated crystals. The isolated crystals display a slightly smaller cell which exactly matches that observed from the structure determination of BmCPV1 obtained with isolated crystals (Coulibaly *et al.*, 2007 ▶). The isolated crystals also display a greater variance in cell length and mosaicity. A clear benefit of the *in cellulo* method is the reduction in crystal handling, which minimizes manual effort and preserves the sample in a favourable and stable environment, as suggested by the unit-cell parameter observations. Additionally, we observe that the process of sample isolation and purification is more likely to produce aggregations of crystals and the complications of recording diffraction from multiple lattices.

Koopmann and coworkers highlighted the potential for *in vivo*-grown crystals and serial crystallography at an XFEL source in structural biology (Koopmann *et al.*, 2012 ▶). Our study makes a logical progression in showing that sample purification and isolation are not necessary and that samples can be directly addressed within the cells that produced them. At the same time, the method becomes more generally applicable with the demonstration that it can be achieved on a microfocus synchrotron beamline. It should be noted that here, in comparison to the cathepsin B example, a larger diffracting volume has been examined (∼100 *versus* ∼10 µm^2^) and with a lower solvent content (24 *versus* 61%). In terms of the protein volume contributing to Bragg spots, these together represent a factor of 20 and hence an expected factor of 20^1/2^ in signal to noise. In practice, signal to noise is comparable, at 11.9 for the cathepsin and 10.4 for the *in cellulo* CPV, although in the highest resolution bin of the cathepsin data the 〈*I*/σ(*I*)〉 is 2.1, whilst at this resolution the value for CPV is 13.5. This reflects the higher average *B* factor reported for cathepsin B (47.5 Å^2^) compared with the *in cellulo* polyhedrin reported here (6.9 Å^2^). Very many more crystals were used in the XFEL serial crystallography experiment, with 178 875 contributing to the final structure and ∼10^8^ expended (Redecke *et al.*, 2013 ▶), compared with the 20 crystals contributing to the final structure of the polyhedrin, for which less than 100 insect cells were required in total. We estimate that ∼60 crystals would be needed to provide similar completeness for the most common space group for proteins, *P*2_1_2_1_2_1_ (extrapolated from Fry *et al.*, 1999 ▶). Furthermore, improvements in the sample environment, reduction in air scatter and improvements in beam collimation might provide an order of magnitude improvement in signal to noise for such small crystals at microfocus synchrotron sources, while continued reductions in storage-ring electron-beam emittance and improved beam-focusing capabilities, potentially coupled to flux gains with the use of pink X-ray beams, will further enhance the scope of this method at third-generation light sources. Certainly, the workflow described here demonstrates how the identification, collection and merging of data from multiple micrometre-scale crystals is becoming a more routine and rapid avenue for structure determination. Opportunities remain to increase the throughput of samples. One aspect is sample preparation; we expect that by working at a higher concentration of cells the grid-scan hit rate could be increased from ∼2% to perhaps 6%. With the current workflow it would then be feasible to collect data from some 960 crystals in 20 h, suggesting that a useful data set could be collected from more weakly diffracting samples in less favourable space groups in a typical allocation of user beam time. Furthermore, the workflow could be streamlined, for example, by automatic detection of grid scan hits to drive data collections at the corresponding positions and by closer linking of the data integration and merging. Ultimately, we anticipate that automated data acquisition and processing for structure determination from *in vivo* produced microcrystals will be achieved.

## Supplementary Material

PDB reference: OpbrCPV18, *in cellulo*, 4otv

PDB reference: isolated, 4ots

## Figures and Tables

**Table 1:** Data-processing statistics Values in parentheses are for the highest resolution shell.

	OpbrCPV18, *in cellulo*	OpbrCPV18, isolated
Wavelength (Å)	0.9686	0.9686
No. of crystals used	20	20
Resolution range (Å)	27.55–1.70 (1.73–1.70)	27.47–1.70 (1.74–1.70)
Space group	*I*23	*I*23
Unit-cell parameter (Å)	*a* = 103.04 [σ = 0.097†]	*a* = 102.79 [σ = 0.153†]
Total No. of reflections	66645 (507)	191083 (1262)
Unique reflections	18161 (412)	19343 (660)
Mosaicity (°)	0.034 [σ = 0.0073†]	0.051 [σ = 0.031†]
Multiplicity	3.7 (1.2)	9.9 (1.9)
〈*I*/σ(*I*)〉	10.4 (2.2)	11.6 (2.5)
Completeness (%)	90.5 (39.2)	97.2 (63.3)
*R*_meas_	0.19 (0.52)	0.185 (0.331)
CC_1/2_	0.978 (0.665)	0.992 (0.809)

† Across all crystals as calculated by *XDS*.

**Table 2:** Refinement statistics Values in parentheses are for the highest resolution shell.

	OpbrCPV18, *in cellulo*	OpbrCPV18, isolated
Resolution range (Å)	27.55–1.70 (1.79–1.70)	27.47–1.70 (1.79–1.70)
No. of reflections	17200 (588)	19327 (2322)
No. of non-H atoms	2314	2307
*R*	0.122 (0.206)	0.100 (0.156)
*R*_free_	0.179 (0.293)	0.132 (0.190)
R.m.s.d., bond lengths (Å)	0.006	0.006
R.m.s.d., bond angles (°)	1.171	1.169

## Footnotes

1 As calculated from a two-sample t-test (*p* < 0.05 can be deemed significant)
